# Supplementary material for: Are carbon emissions trading and green financial instruments synergistic? -Comprehensive quantitative research based on content analysis
Source: PLoS One. 2024 Mar 7;19(3):e0298601. doi: 10.1371/journal.pone.0298601 (PMC10919601; doi:10.1371/journal.pone.0298601)
Supplement: S1 Data — (ZIP) [file pone.0298601.s001.zip › Data/地级市Z_F工作报告.zip!/╕ú╜¿/╚2├≈╩╨/└┤╫╘╣1⁄2╓┌║┼í╛┬φ┐╦╩2╛▌═°í┐_2007.docx]

勇当前锋科学发展推进海峡西岸纵深经济区又好又快发展

今后五年政府工作总体要求

　 市长张健在市十一届人大一次会议上作政府工作报告时强调，今后五年，经济全球化和国际产业调整转移，为我们利用两个市场、两种资源拓展了空间；中央支持海峡西岸发展，国家部委出台扶持政策，为我们融入大局、加快发展提供了机遇；省委、省政府确立三明在海峡西岸纵深推进的前锋定位，省委卢展工书记对三明发展提出“前锋、基地、枢纽、支撑”的要求，为我们构建比较完善的基础设施、产业、城市体系创造了条件。为此，我市力争更好更快地实现今后五年政府工作总体要求。

　 今后五年政府工作总体要求是：以邓小平理论和“三个代表”重要思想为指导，认真贯彻党的十六届三中、四中、五中、六中全会精神，落实省第八次党代会和市第七次党代会部署，以科学发展观统领经济社会发展全局，深入实施“工业立市、生态兴市、科教强市”战略，重在持续，重在提升，重在运作，重在实效，着力构建海峡西岸重要综合交通枢纽，着力建设社会主义新农村，着力壮大区域经济和中心城市，着力推进“和谐三明”建设，到2011年，综合实力实现新突破，发展后劲得到新提升，和谐社会呈现新局面，人民生活再上新水平，在海峡西岸建设中实现加快崛起和更大跨越。

各项事业全面进步重要领域取得突破

　 市长张健作政府工作报告时指出，自2002年12月本届政府组成以来，我市全面贯彻落实科学发展观，主动融入海峡西岸建设，经过全市人民共同努力，经济持续较快增长，各项事业全面进步，一些重要领域取得突破。

　 张健说，交通基础设施实现跨越。2003—2006年交通建设投资123.73亿元，超过1949—2002年的总和，公路投资居全省第一；高速公路通车里程全省第三，福银高速公路三明段建成通车，泉三、永武高速公路三明段动工，永宁高速公路控制性工程预计年内开工;四年完成国、省道干线和重要经济公路建设改造665.28公里；硬化农村公路4350.2公里，居全省第二，硬化路面通乡率达100%，通村率由2002年的30%提高到2006年的81.9%；三明机场开工续建；闽中快速通道向莆铁路控制性工程预计一季度动工；海峡西岸三明综合交通枢纽规划启动。产业集群快速成长。冶金产业集群跻身全省13个产值超百亿元的产业集群行列，林产工业产值全省第一，我市列入全省生物医药产业重点区域和重型汽车生产基地。林业改革率先推进。集体林权制度改革走在全国前列，设立全国唯一的海峡两岸（三明）现代林业合作实验区。品牌战略取得突破。泰宁地质公园进入世界地质公园行列，“铙山”商标被认定为中国驰名商标，双轮牌轮胎硫化机、永林蓝豹牌中密度纤维板成为中国名牌产品，建宁白莲获得地理标志产品保护。外贸出口增势强劲。2006年外贸出口总额5.38亿美元，是2002年的4.56倍，年均增长46.1%。教育、体育事业亮点凸显。连续四年高考上线率和录取率保持全省前列，三明学院、福建水利电力职业技术学院和三明职业技术学院相继成立。我市运动员在第十五届亚运会上实现金牌零的突破。和谐社会建设取得新成效。我市再次被授予“全国创建文明城市工作先进城市”称号，首次进入全国社会治安综合治理优秀地市行列，沙溪两岸生态环境整治工程获“中国人居环境范例奖”。
